# Genomic investigation refutes record of most diverged avian hybrid

**DOI:** 10.1002/ece3.9689

**Published:** 2023-01-06

**Authors:** James M. Alfieri, Taryn Johnson, Anna Linderholm, Heath Blackmon, Giridhar N. Athrey

**Affiliations:** ^1^ Interdisciplinary Program in Ecology and Evolutionary Biology Texas A&M University College Station Texas USA; ^2^ Department of Biology Texas A&M University College Station Texas USA; ^3^ Department of Poultry Science Texas A&M University College Station Texas USA; ^4^ Department of Anthropology Texas A&M University College Station Texas USA; ^5^ Centre for Palaeogenetics, Department of Geological Sciences Stockholm University Stockholm Sweden

**Keywords:** avian, divergence, genomics, hybridization, species identification

## Abstract

The most diverged avian hybrid that has been documented (*Numida meleagris* × *Penelope superciliaris*) was reported in 1957. This identification has yet to be confirmed, and like most contemporary studies of hybridization, the identification was based on phenotype, which can be misleading. In this study, we sequenced the specimen in question and performed analyses to validate the specimen's parentage. We extracted DNA from the specimen in a dedicated ancient DNA facility and performed whole‐genome short‐read sequencing. We used BLAST to find Galliformes sequences similar to the hybrid specimen reads. We found that the proportion of BLAST hits mapped overwhelmingly to two species, *N. meleagris* and *Gallus gallus*. Additionally, we constructed phylogenies using avian orthologs and parsed the species placed as sister to the hybrid. Again, the hybrid specimen was placed as a sister to *N. meleagris* and *G. gallus*. Despite not being a hybrid between *N. meleagris* and *P. superciliaris*, the hybrid still represents the most diverged avian hybrid confirmed with genetic data. In addition to correcting the “record” of the most diverged avian hybrid, these findings support recent assertions that morphological and behavioral‐based identifications of avian hybrids can be error‐prone. Consequently, this study serves as a cautionary tale to researchers of hybridization.

## Graphical abstract

In this article, Alfieri and colleagues revisit the identification of a historic avian hybrid, and using modern genetic techniques, correct the identification, while also correcting the record of the most diverged avian hybrid.

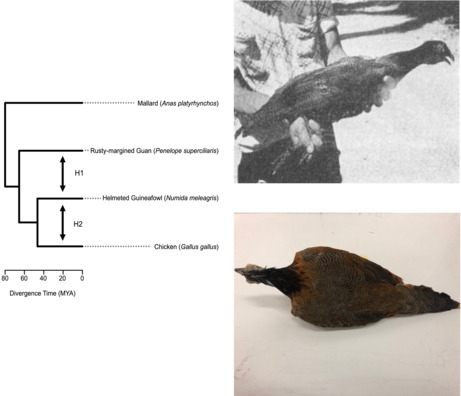

## 1 INTRODUCTION

Hybridization between species is an important evolutionary event that generates genomic and phenotypic diversity and affects speciation (Moran et al., 2021). Most contemporary hybridization records rely on phenotypic observations, which can be controversial (Hill et al., 2021; Justen et al., 2021; Justyn et al., 2020, 2022; Minor et al., 2022). Genetic approaches, such as whole‐genome sequencing, remain the gold standard for validating hybridization events (Ottenburghs, 2021). One specific hybridization record by (Ruschi & Amadon, 1959) is particularly pertinent to the ongoing discussion on the validity of phenotypic observations of hybrids, even when made by trained ornithologists. If correct, this represents the most diverged hybridization event documented in Aves. In this study, we used genomic data to illuminate the true origins of this historic hybrid.

**FIGURE 1:**
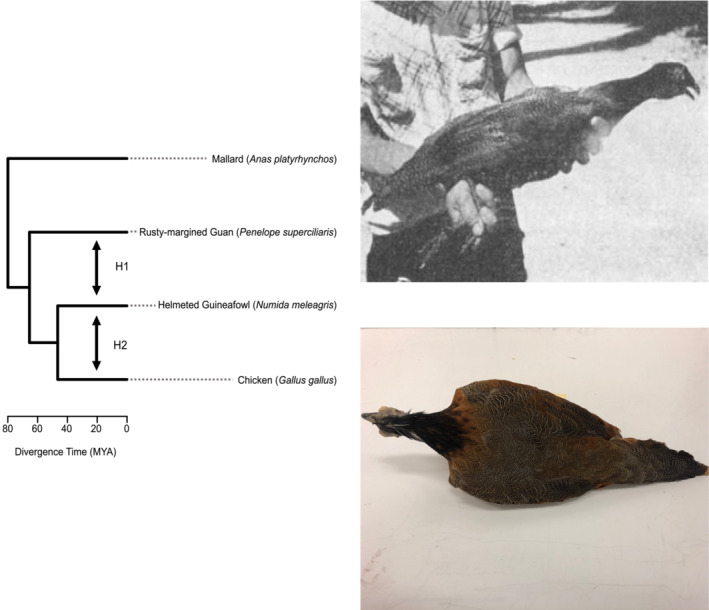
Competing hypotheses for the parentage of the avian hybrid. H1 represents the original identification (*Penelope superciliaris* × *Numida meleagris*). H2 represents the revisited identification (*Gallus gallus* × *Numida meleagris*). Top right image is the original 1956 photograph of the hybrid taken by Dean Amadon. Bottom right image is a 2019 photograph of the specimen by Peter Capainolo and is copyrighted by the American Museum of Natural History with permission to reuse in this publication.

Although there are anecdotal reports of interfamilial hybrids involving the guan family (Family Cracidae, Order Galliformes; Aquarone, 1869; Dresser, 1866; Goodfellow, 1902), only one was documented substantially. Dean Amadon, Chairman of the Department of Ornithology at the American Museum of Natural History in New York City, New York, USA, reported on an extreme hybrid (Ruschi & Amadon, 1959) at the Pan‐African Ornithological Congress held in Livingstone, South Africa, in 1957. The putative hybrid was a cross between *Penelope superciliaris* (rusty‐margined guan) and *Numida meleagris* (helmeted guineafowl). He described a 2‐year‐old bird in possession of the Brazilian Ornithologist Augusto Ruschi, who had acquired the individual from a farm in São Pedro, Brazil, in 1956. Amadon's stature in the field lent credibility to this observation but did not prevent controversy. The French ornithologist Jacques Berlioz claimed that such a mating would be impossible due to differences in cloacal structures and suggested that the suspected parentage was probably wrong (Ruschi & Amadon, 1959). After Ruschi sent the preserved skin to the American Museum of Natural History in 1957, Amadon reconsidered his opinion, suggesting that the specimen could be a hybrid between the *N. meleagris* and *Gallus gallus* (chicken), perhaps cautioned by the extreme divergence of the parents. He suggested that further investigation was warranted to settle the question of the specimen's parentage (Figure 1; Delacour & Amadon, 1973).

Hybridization of exceptionally diverged species represents “points of failure” in isolating mechanisms, emphasizing the significance of identifying the actual parents for understanding divergence and reproductive incompatibilities (Edmands, 2002). The putative parents of the hybrid specimen, *P. superciliaris* and the *N. meleagris*, are estimated to have diverged ~65 MYA (Kumar et al., 2017). Other less‐diverged but still remarkable avian examples include *N. meleagris* × *G. gallus* hybrids (diverged ~47 MYA) and *Cardinalis cardinalis* (northern cardinal) × *Paroaria coronata* (red‐crested cardinal) hybrids (diverged ~38 MYA; McCarthy, 2006).

The maximum documented divergence of hybridizing species varies among clades. In mammals, for example, the most diverged hybrid is between *Camelus dromedarius* (dromedary camel) and *Lama guanicoe* (guanaco), estimated to have diverged ~20 MYA (Skidmore et al., 1999). The most diverged fish hybrid was produced in the laboratory and is between *Polyodon spathula* (American paddlefish) and *Acipenser gueldenstaedtii* (Russian sturgeon), estimated to have diverged ~150 MYA (Káldy et al., 2020). The most diverged reptile hybrid, confirmed with genetic data, is between *Eretmochelys imbricata* (hawksbill sea turtle) and *Chelonia mydas* (green sea turtle), estimated to have diverged ~47.5 MYA (Brito et al., 2020). These examples highlight the possibility that the putative *N. meleagris* × *P. superciliaris* hybrid is the most extreme hybrid among birds but not all vertebrates. The viability of these hybrids warrants investigation as it is a critical threshold that organisms must reach to halt gene flow.

In this study, we investigated the parentage of this hybrid using whole‐genome sequencing. We find that the initial determination of the parent species was incorrect and that the hybrid is the offspring of *G. gallus* and *N. meleagris* (estimated divergence time 47 MYA). Notwithstanding the incorrect identification in 1957, this hybrid represents a confirmed case of extreme divergent hybridization in birds. Additionally, this study supports recent assertions that morphological and behavioral‐based identifications of avian hybrids are error‐prone (Ottenburghs, 2021) and that genetic determination is the gold standard. Our study highlights the challenges of correctly assigning parentage to hybrids, even in captive environments.

## 2 MATERIALS AND METHODS

### 2.1 Sample and DNA extraction

We obtained a toe clipping of the putative *N. meleagris* × *P. superciliaris* hybrid from the American Museum of Natural History Ornithology Collection (SKIN 775723) in April 2019. DNA extraction was performed in a dedicated ancient DNA facility at Texas A&M University, in the Bioarchaeology and Genomics Laboratory. We partitioned the tissue into three technical replicates and performed extractions with negative controls. We washed the specimen with bleach to remove exogenous DNA and used a modified phenol–chloroform DNA extraction protocol, skipping the bead purification steps to preserve yields (Tsai et al., 2020). The negative controls did not contain DNA as ascertained by Qubit fluorometric quantification (ThermoFisher Scientific).

### 2.2 Library preparation, sequencing, and QC

Library preparation and sequencing were performed at the Texas A&M Institute for Genome Sciences and Society (College Station, TX USA). Briefly, libraries were prepared using the Illumina Nextera XT library preparation method for 150‐bp paired‐end reads and sequenced on an Illumina NovaSeq 6000 on one lane of an S4 flow cell.

We assessed sequence quality with FastQC (Andrews, 2010) and trimmed raw reads to have a minimum Phred score of 30 and length of 30 with Trim Galore (Krueger, 2012). Additionally, we removed reads with >30 masked bases. Overall, our sequencing yielded ~800,000,000 quality‐filtered reads, with an average length of 90. Assuming a genome size of 1.2 Gb (approximate haploid genome size of *G. gallus*), resulting in a coverage of ~60×.

### 2.3 Bioinformatics and analysis

We first performed a local BLAST search to identify the hybrid's parent species (Altschul et al., 1990). Our BLAST database contained genome sequences from 17 Galliformes and one Anseriformes outgroup (Appendix 1). The database includes species representing all families within Galliformes, including the putative parent species or species closely related to them (e.g., we include *P. pileata* instead of *P. superciliaris* due to the availability of assemblies). In order to account for differences in genome size and contiguity between genome assemblies, we partitioned each species' genome assembly into 100‐basepair fragments, followed by randomly sampling 1,000,000 fragments of each genome (~10% of each species' genome). These reduced genomes were used to construct the local BLAST database. We then used our quality‐filtered hybrid reads as queries using our BLAST database and default parameters. We parsed the results to retain the best subject match (lowest *E*‐value) per query, discarding queries where two or more subject species tied for the lowest *E*‐value. Additionally, we constructed a separate BLAST database containing only the mitochondrial genomes for 28 Galliformes species and one Anseriformes species to identify the maternal parent species with the same approach (Appendix 2).

In addition to the BLAST searches, we constructed phylogenies of orthologs. We used OrthoFinder to obtain the amino acid sequences of orthologs shared between six representative Galliformes and one Anseriformes species: *Meleagris gallopavo*, *Colinus virginianus*, *Numida meleagris*, *Gallus gallus*, *Phasianus colchicus*, *Struthio camelus*, and *Anas platyrhynchos* (Emms & Kelly, 2019). We selected these species because they had annotated genomes and represented a diversity of families across Galliformes. We identified 8063 single‐copy orthologs shared between these taxa. We used the gene ID output from OrthoFinder to parse the *Anas platyrhynchos* CDS fasta file and obtain the nucleotide sequence corresponding to the shared single‐copy orthologs. We then obtained short‐read sequence data for 17 Galliformes and aligned these to the *Anas platyrhynchos* shared single‐copy orthologs, after performing quality trimming with FastQC and Trim Galore (Andrews, 2010; Krueger, 2012). The short‐read dataset contains species representing all families within Galliformes, including the putative parent species or species closely related to them (e.g., we include *Penelope pileata* instead of *P. superciliaris* due to the availability of assemblies). We used default settings to align the quality‐trimmed 17 Galliformes sequences and the one hybrid dataset to the *Anas platyrhynchos* single‐copy ortholog sequences with bwa aligner (Li & Durbin, 2009). Using bcftools, we built consensus sequences of the mapped reads, using the alternate allele for heterozygous genotypes and the reference allele for homozygous genotypes (Danecek et al., 2021). This process resulted in 8063 sequences aligned to the *Anas platyrhynchos* outgroup. After this, we performed a multiple sequence, multispecies alignment for the 8063 sequences using MAFFT default settings (Katoh & Standley, 2013). We built phylogenies for these alignments using the default settings of IQ‐TREE2 (Minh et al., 2020). Finally, we parsed the 8063 ortholog phylogenies to extract the sister species of the hybrid using R Packages ape, phytools, and diverge (Anderson & Weir, 2022; Paradis & Schliep, 2019; Revell, 2012).

## 3 RESULTS

**FIGURE 2:**
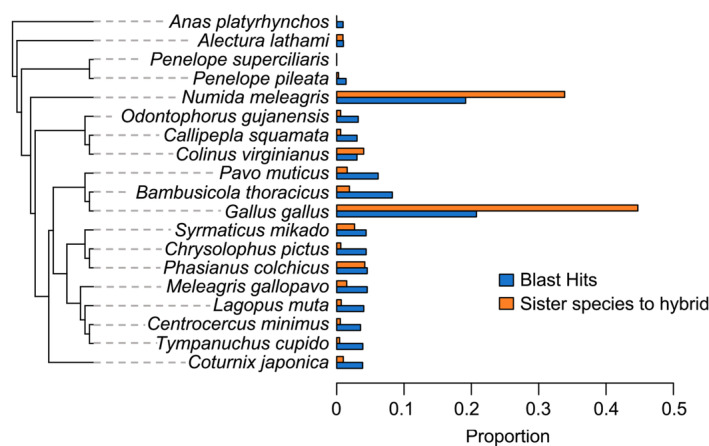
Proportion of unique, best hits from BLAST analysis (blue) and proportion of phylogenies placing a species as sister to the hybrid (orange). Although *Penelope superciliaris* was one of the hypothesized parent species, there are no genomic data available. Nonetheless, we have included the species in the phylogeny for visualization purposes.

We obtained ~400,000,000 BLAST high‐scoring pairs for our species identification analysis, of which ~170,000,000 represented unique, low *E*‐value hits. The hits were overwhelmingly concentrated on two species: *N. meleagris* (20% of total hits) and *G. gallus* (20% of total hits; Figure 2). In our mitochondrial analysis, we obtained ~18,000,000 BLAST high‐scoring pairs, of which ~3,000,000 represented unique, best hits. 99.9% of mitochondrial hits were to *N. meleagris*.

In our ortholog analysis, of the 8063 phylogenies, we found 5910 placed a single taxa sister to the hybrid. Of these trees, 45% placed *G. gallus* as sister to the hybrid, while 34% of these trees placed *N. meleagris* as sister to the hybrid (Figure 2). By contrast, only 0.2% of these trees placed *P. pileata* (the representative cracid in our dataset) as sister to the hybrid.

## 4 DISCUSSION

We found that the initial identification of the hybrid (*P. superciliaris* × *N. meleagris*) was incorrect. Our data suggest that the specimen was actually a hybrid between *G. gallus* and *N. meleagris*, estimated to have diverged ~47 MYA. Although this hybrid is commonly reported in aviaries (Beddard, 1899; Guyer, 1909; Mathis et al., 1983; McCarthy, 2006; Morton, 1847), it still represents a case of extreme hybridization and, indeed, is currently the most diverged avian hybrid confirmed using genetic data. It is important to note that this species pair is equal in divergence to other extreme hybrids involving *N. meleagris* produced in captivity, for example, crosses with *Pavo cristatus* (Hanebrink et al., 1973). Identification of the true progenitors of this hybridization record is important because any comparative analyses of hybridization will only be as accurate as the data they begin with. Additionally, our results question the veracity of hybrid identifications based on phenotypic observations alone. Even with the specimen in hand and accompanied by detailed behavioral observations and provenance, trained ornithologists were unable to assign parentage accurately to this hybrid. While there are other possibilities in this case, including an F2 backcross with one or other parental species, we can rule them out in favor of the inference that this specimen is a Gallus × Numida F1 hybrid. These reasons are:

- We find that the BLAST hits to *N. meleagris* and *G. gallus* are roughly equal (~20%), which is consistent with the hybrid being an F1. Additionally, the BLAST analysis revealed that neither of the most likely parents showed greater than 50% of BLAST hits. As our mtDNA analysis supports *N. meleagris* as the dam, we would expect any F1 backcrossed with *N. meleagris* to be >50% of BLAST hits to the *N. meleagris* genome. We base this on the expectation that any backcross F2 individual would likely show ~75% of BLAST hits to the backcrossed parent. We failed to observe this pattern.
- If a backcross F2 had been born in captivity, this would have been an even more remarkable occurrence from the perspective of Amadon and Ruschi. Their failure to raise such a possibility or allude to other such occurrences in Brazil suggests the hybrid is not F2. Wrong as they turned out to be regarding the parentage of the hybrid, they would not have neglected to consider the F2 if was plausible. Additionally, while there are many records of *G. gallus* × *N. meleagris* crosses, there are no records of a fertile hybrid between these species.

Therefore, even with imperfect genome assemblies, the evidence for an F1 hybrid is overwhelming.

There are multiple proposed explanations for why certain species pairs can produce viable hybrid individuals despite tens of millions of years of divergence. One hypothesis why some species can hybridize despite large divergence times is that the species pair diverged allopatrically with no ongoing secondary contact, decreasing the strength of selection for reproductive isolation (reinforcement). Another hypothesis of why certain species are able to hybridize across large divergence times, while others are not, is that the rate of evolution of regulatory genes is slower in some clades than in others (Prager & Wilson, 1975). A related train of thought is that some stages of development are more prone to hybrid dysfunction than other stages due to the ontogenetic timing of gene expression (Cutter & Bundus, 2020). Perhaps the effects of mutations that disrupt development are less severe in species able to hybridize across large divergence times. Finally, it is possible that evolution associated with the process of domestication plays a role in the probability of successfully hybridizing. This could be a function of both the likelihood of a hybrid being viable and the likelihood of a hybridization attempt occurring. Indeed, many species capable of hybridizing across large divergence times have a history of domestication such as chicken, guineafowl, sturgeon, camels, and llamas among others. However, these examples may just represent a biased sample, as domesticated animals are more closely associated with human observers than wild species. Using a comparative framework of hybridization ability across a range of divergence times may help to evaluate these hypotheses.

Recently, there has been disagreement over the use of community science datasets, such as Ebird, to measure individual and species rates of hybridization in the wild (Hill et al., 2021; Justen et al., 2021; Justyn et al., 2020, 2022; Minor et al., 2022). Justyn et al. (2020) used Ebird to quantify rates of hybridization, acknowledging that citizen scientists may both underreport and overreport hybridization events. Justen et al. (2021) argue that citizen scientists underreport hybrids compared with experts partly due to the difficulty of determining hybrids through phenotypic observation, supported by a comparison of species hybridization rates in Ebird to those obtained from Birds of North America. In response, Hill et al. (2021) clarify their interpretation of the term “hybrid” as “an individual with a phenotype that is intermediate between two parental species.” While our case study is not directly related to determining individual or species rates of hybridization, it is germane to the issue of relying on observations of “intermediate phenotype” to assess hybrid parentage. We argue that the use of citizen science databases (which rely on phenotypic observations made by nonexperts) to correctly identify parentage should be done with caution and results should be interpreted as preliminary at best.

To summarize, we solved a 64‐year‐old mystery over the parentage of a unique avian hybrid individual. Using whole‐genome analyses, we determined that the parents of the hybrid were the frequently observed, yet still extremely diverged species pair, *G. gallus* (sire) and *N. meleagris* (dam). This result means that there is still no conclusive evidence that birds in the family Cracidae are capable of interfamilial hybridization. Our study serves as a cautionary tale for researchers relying on phenotypic observations to determine hybrid parentage. Finally, our study corrects a historical record in the literature, providing for more accurate analyses of hybridization and divergence relationships in a comparative framework.

## AUTHOR CONTRIBUTIONS

**James M. Alfieri:** Conceptualization (equal); data curation (equal); formal analysis (equal); funding acquisition (equal); investigation (equal); methodology (equal); project administration (equal); visualization (equal). **Taryn Johnson:** Methodology (equal); project administration (equal); supervision (equal); writing – review and editing (equal). **Anna Linderholm:** Methodology (equal); resources (equal); supervision (equal); writing – review and editing (equal). **Heath Blackmon:** Conceptualization (equal); data curation (equal); formal analysis (equal); funding acquisition (equal); investigation (equal); methodology (equal); project administration (equal); resources (equal); software (equal); supervision (equal); visualization (equal); writing – original draft (equal); writing – review and editing (equal). **Giridhar N. Athrey:** Conceptualization (equal); data curation (equal); formal analysis (equal); funding acquisition (equal); investigation (equal); methodology (equal); project administration (equal); resources (equal); software (equal); supervision (equal); visualization (equal); writing – original draft (equal); writing – review and editing (equal).

## CONFLICT OF INTEREST

The authors declare no conflict of interest.

## Data Availability

The sequencing data of the hybrid specimen have been deposited to SRA under BioSample Accession SAMN32101650 and BioProject ID PRJNA909752.

## APPENDIX 1 Species used for BLAST and ortholog analysis

**Table:**

Family	Species	GenBank assembly accession (BLAST)	Approximate sequence length (Gb)	SRA numbers (Orthologs)
Anatidae	*Anas platyrhynchos*	GCA_015476345.1	1.2	NA
Megapodiidae	*Alectura lathami*	GCA_013399715.1	1.0	SRR10019965
Cracidae	*Penelope pileata*	GCA_013396635.1	1.0	SRR9994352
Numididae	*Numida meleagris*	GCA_002078875.2	1.0	SRR12042133
Odontophoridae	*Odontophorus gujanensis*	GCA_013399175.1	0.98	SRR9946998
Odontophoridae	*Callipepla squamata*	GCA_002218305.1	1.0	SRR3901028
Odontophoridae	*Colinus virginianus*	GCA_008692595.2	0.95	SRR4305253
Phasianidae	*Gallus gallus*	GCA_000002315.5	1.1	SRR9967586
Phasianidae	*Pavo muticus*	GCA_016647715.1	1.1	SRR12223790
Phasianidae	*Chrysolophus pictus*	GCA_003413605.1	1.0	SRR7647148
Phasianidae	*Bambusicola thoracicus*	GCA_002909625.1	1.0	SRR6466369
Phasianidae	*Syrmaticus mikado*	GCA_003435085.1	1.1	SRR5666076
Phasianidae	*Phasianus colchicus*	GCA_004143745.1	1.0	SRR11679531
Phasianidae	*Meleagris gallopavo*	GCA_000146605.4	1.1	ERR5191642
Phasianidae	*Lagopus muta*	GCA_004320205.1	1.0	SRR8393387
Phasianidae	*Centrocercus minimus*	GCA_005890655.1	1.0	SRR8863302
Phasianidae	*Tympanuchus cupido*	GCA_001870855.1	0.98	SRR8616843
Phasianidae	*Coturnix japonica*	GCA_001577835.2	0.90	SRR13858145

## APPENDIX 2 Species used for mitochondrial BLAST analysis

**Table:**

Family	Scientific name	Mitochondrial accession
Megapodiidae	*Alectura lathami*	GCA_013399715.1
Phasianidae	*Bambusicola thoracicus*	GCA_002909625.1
Odontophoridae	*Callipepla squamata*	GCA_002218305.1
Phasianidae	*Centrocercus minimus*	GCA_005890655.1
Phasianidae	*Chrysolophus pictus*	GCA_003413605.1
Odontophoridae	*Colinus virginanus*	GCA_008692595.2
Phasianidae	*Coturnix japonica*	AP003195.2
Phasianidae	*Gallus gallus*	GCA_016699485.1
Phasianidae	*Lagopus muta*	GCA_004320205.1
Phasianidae	*Lyrurus tetrix tetrix*	GCA_000586395.1
Phasianidae	*Meleagris gallopavo*	GCF_000146605.3
Numididae	*Numida meleagris*	GCF_002078875.1
Odontophoridae	*Odontophorus gujanensis*	GCA_013399175.1
Phasianidae	*Pavo cristatus*	GCA_005519975.1
Phasianidae	*Pavo muticus*	GCA_016647715.1
Cracidae	*Penelope pileata*	GCA_013396635.1
Phasianidae	*Phasianus colchicus*	GCF_004143745.1
Phasianidae	*Syrmaticus mikado*	GCA_003435085.1
Phasianidae	*Tympanuchus cupido*	GCA_001870855.1
